# Assessment of Artificial MiRNA Architectures for Higher Knockdown Efficiencies without the Undesired Effects in Mice

**DOI:** 10.1371/journal.pone.0135919

**Published:** 2015-08-18

**Authors:** Hiromi Miura, Hidetoshi Inoko, Masafumi Tanaka, Hirofumi Nakaoka, Minoru Kimura, Channabasavaiah B. Gurumurthy, Masahiro Sato, Masato Ohtsuka

**Affiliations:** 1 Department of Molecular Life Science, Division of Basic Medical Science and Molecular Medicine, School of Medicine, Tokai University, 143 Shimokasuya, Isehara, Kanagawa 259–1193, Japan; 2 Division of Human Genetics, Department of Integrated Genetics, National Institute of Genetics, Yata 1111, Mishima, Shizuoka 411–8540, Japan; 3 Mouse Genome Engineering Core Facility, Department of Genetics, Cell Biology and Anatomy, University of Nebraska Medical Center, Omaha, Nebraska, 68198, United States of America; 4 Section of Gene Expression Regulation, Frontier Science Research Center, Kagoshima University, 1-21-20 Korimoto, Kagoshima, Kagoshima 890–0065, Japan; 5 The Institute of Medical Sciences, Tokai University, 143 Shimokasuya, Isehara, Kanagawa 259–1193, Japan; Rutgers—New Jersey Medical School, UNITED STATES

## Abstract

RNAi-based strategies have been used for hypomorphic analyses. However, there are technical challenges to achieve robust, reproducible knockdown effect. Here we examined the artificial microRNA (amiRNA) architectures that could provide higher knockdown efficiencies. Using transient and stable transfection assays in cells, we found that simple amiRNA-expression cassettes, that did not contain a marker gene (−MG), displayed higher amiRNA expression and more efficient knockdown than those that contained a marker gene (+MG). Further, we tested this phenomenon *in vivo*, by analyzing amiRNA-expressing mice that were produced by the pronuclear injection-based targeted transgenesis (PITT) method. While we observed significant silencing of the target gene (eGFP) in +MG hemizygous mice, obtaining −MG amiRNA expression mice, even hemizygotes, was difficult and the animals died perinatally. We obtained only mosaic mice having both “−MG amiRNA” cells and “amiRNA low-expression” cells but they exhibited growth retardation and cataracts, and they could not transmit the –MG amiRNA allele to the next generation. Furthermore, +MG amiRNA homozygotes could not be obtained. These results suggested that excessive amiRNAs transcribed by −MG expression cassettes cause deleterious effects in mice, and the amiRNA expression level in hemizygous +MG amiRNA mice is near the upper limit, where mice can develop normally. In conclusion, the PITT-(+MG amiRNA) system demonstrated here can generate knockdown mouse models that reliably express highest and tolerable levels of amiRNAs.

## Introduction

RNA interference (RNAi)-mediated gene silencing is a useful approach for rapidly obtaining hypomorphic phenotypes by repressing selected gene. Many different strategies are used for RNAi-mediated gene silencing that involve the expression of sequences such as short interference RNA (siRNA), short hairpin RNA (shRNA) or artificial microRNA (amiRNA). These sequences are designed to include the complementary sequence for a target mRNA with which they bind in RNA-induced silencing complex that will repress target gene expression (knockdown) both *in vitro* and *in vivo* [1, 2]. Because RNAi-mediated gene silencing is easier to use and gene function can be more rapidly analyzed compared to knockout techniques until recently when CRISPR/Cas9 system became popular, it has been widely used for analyzing a gene’s function, for genome-wide genetic screening and also for gene therapy.

In order to assess the gene function reliably and satisfactorily, it is important to achieve sufficient knockdown of the target gene that can elicit a phenotype for further analysis. Several *in vitro* approaches have been employed by researchers to improve the efficiency of RNAi-mediated gene silencing. For example, McBride et al. screened shRNAs *in vitro* to select the most effective from among many designed shRNAs [3]. Some groups optimized hairpin structures or miRNA backbones that could improve knockdown potency [4, 5]. Another group used multiple amiRNA in a single transcript [6]. However, such strategies have not been systematically evaluated using *in vivo* models. In contrast, it is also reported that high shRNA expression levels can result in severe toxicity in some tissues (e.g., liver, central nervous system, and heart) and/or lethality in mice, rats, and dogs [3, 7]. These potential adverse effects of *in vivo* RNAi might be alleviated by using shRNA embedded within a natural miRNA backbone (also known as artificial microRNA/amiRNA) although *in vivo* toxicity of amiRNA architectures is not well studied.

The *in vivo* knockdown effects using RNAi have often been evaluated by administering siRNA or establishing transgenic (Tg) mice by using plasmids or virus vectors that contain shRNA- or amiRNA-expression cassettes [8]. However, the knockdown levels in mice obtained with these methods vary and are often not reproducible due to transient inhibition by siRNA or unreliable expression of shRNA/amiRNA in those Tg mice generated by random integration-based transgenesis [8, 9]. Although these pitfalls can be circumvented by using targeted Tg mice generated via ES cell targeting [10], the ES cell-based methods are laborious, expensive and time-consuming.

We recently developed a novel system to generate Tg mice that we called Pronuclear Injection-based Targeted Transgenesis (PITT), for targeted insertion of a single-copy of a transgene into a predetermined genomic locus, such as *Gt(ROSA)26Sor* (*Rosa26*) [11]. Because transgenes can be delivered to zygotes via pronuclear injection similar to conventional transgenesis methods, rapid, simple generation of Tg mice is achieved through PITT. Using this method, we have generated a series of Tg mouse lines that express a gene of interest driven by a ubiquitous CAG promoter. Using this technique, strong, reproducible and stable transgene expression can be achieved. We also demonstrated that this method can be used for generating knockdown mice [11], although, in our previous study, we had not characterized whether these mice exhibit reproducible, stable knockdown efficiencies.

In this study, we compared the efficiency of gene silencing of amiRNA expression cassettes with- or without-marker genes and also demonstrated that our PITT method is suitable for creating amiRNA-expressing mice with highly reproducible knockdown efficiency. We found that the simplest amiRNA-expression cassette that lacked a marker gene exhibited a higher degree of knockdown efficiency both *in vitro* and *in vivo*. The higher amiRNA expression levels (in constructs lacking marker gene) impaired the normal growth and viability of mice. We also demonstrate that marker gene containing amiRNA Tg mice generated using PITT showed higher and desirable levels of knockdown without the toxicity or viability issues.

## Materials and Methods

### Plasmid construction

The vectors required to knockdown eGFP gene were constructed as follows. The amiRNA sequences for targeting eGFP gene (eGFP123 and eGFP419) were designed using BLOCK-iT RNAi designer (Invitrogen, Carlsbad, CA) (S1 Table). The synthetic DNA oligonucleotides (top and bottom) were annealed and cloned into a” BLOCK-iT Pol II miR RNAi Expression Vector with EmGFP” (K4936-00; Invitrogen). Using multi-step cloning, the DNA fragment that spanned each pre-miRNA (5′ miR flanking region - amiRNA sequence - 3′ miR flanking region) was finally cloned into the first intron, last intron, or the 3′ untranslated region (3′ UTR) of the different expression vectors, which resulted in 18 knockdown vectors (No. 1–16, 25, 26 in S2 Table). pT-1, pT-2, and pDre (No. 17–19) were generated by a ligation-based cloning. The codon-optimized *Dre* gene in pDre, which included the “CAG promoter-Dre-polyA” cassette, was synthesized by TaKaRa (Kyoto, Japan), based on the published amino acid sequence of the Dre protein [12]. The vectors (No. 20–26) and some of the components used (tdTomato, eGFP, PB-3′TR, and PB-5′TR) were previously described [11, 13–15]. The *rox* sequence (used in plasmids No. 14–16) was derived from synthesized oligos [12]. The plasmid resources, sequences, and maps are available to the scientific community through Addgene. Information for the expression cassettes for all constructs is shown in S2 Table.

### Cell culture and transfection

ES cells, eGFP-expressing ES cells [11] and E14tg2a [16] or E14.1 [17], were cultured in Dulbecco’s modified Eagle’s medium (Gibco, Grand Island, NY) supplemented with 15% fetal bovine serum, 0.5% penicillin/streptomycin, and 0.01% leukemia inhibitory factor (LIF; Chemicon, Temecula, CA) at 37°C in a humidified atmosphere with 5% CO_2_. Cells were seeded onto 12-well plates (1 × 10^5^ cells/well) or 24-well plates (5.7 × 10^4^ cells/well) one day before transfection. To compare the knockdown efficiencies among the knockdown constructs in ES cells, amiRNA expression vectors (at the same molar amounts of pCAG in control experiments: 1 μg/well in 24-well plates and 2 μg/well in 12-well plates) were transfected into cells using Lipofectamine 2000 (Invitrogen), according to the manufacturer’s protocol, and with or without pG (0.63 μg/well in 24-well plates and 1.25 μg/well in 12-well plates) for eGFP knockdown. For some experiments, pL was co-transfected with these vectors to evaluate transfection efficiencies. After incubation for 48 hours, the cells were observed for fluorescent signals.

To test whether the knockdown efficiency was affected by the presence of a marker gene, eGFP-expressing ES cells were subjected to co-transfection using Lipofectamine 2000 (Invitrogen) with each of the PB-based amiRNA expression vectors (No. 14–16 in S2 Table), and a PB transposase expression vector (pPBase), and/or a *neo* ^*r*^ gene expression PB vector (pPBN) [18, 19]. The eGFP-expressing ES cells had the eGFP-expression cassette (CAG promoter-eGFP-polyA) inserted into the *Rosa26* locus by recombinase-mediated cassette exchange [11]. At 24–48 h after transfection, cells were subjected to selection using G418 (300 μg/ml) for 1–2 weeks. Colonies that were considered to be derived from a single cell were picked and propagated further. These propagated cells were then subjected to Lipofectamine 2000-based transfection with a Dre expression vector (pDre). After about a week, colonies were picked and propagated. eGFP and amiRNA expression in each clone was analyzed by FACS and by real-time PCR, respectively.

### Mice

All mice were maintained in the Center of Genetic Engineering for Human Diseases (CGEHD) animal facility at School of Medicine, Tokai University. C57BL/6J and BDF1 mice were obtained from CLEA Japan, Inc. (Tokyo, Japan). The FLPe Tg mouse strain (C57BL/6-Tg(CAG-FLPe)37Ito/ItoRbrc: RBRC01835) was provided by RIKEN BRC [20], which participates in the National Bio-Resource Project of MEXT, Japan. Each Tg mouse line was previously generated in our facility: AWV^Δex^ (STOCK Gt(ROSA)26Sor<tm9.1(CAG-tdTomato/RNAi:EGFP)Maoh> derived from pAWV for eGFP knockdown), AWK^ex^ (STOCK Gt(ROSA)26Sor<tm11(CAG-RNAi:EGFP)Maoh> derived from pAWK for eGFP knockdown), eGFP Tg (B6.Cg-Gt(ROSA)26Sor<tm2.1(CAG-EGFP)Maoh> derived from pAMF for eGFP expression), and tdTomato Tg (B6.Cg-Gt(ROSA)26Sor<tm6.1(CAG-tdTomato)Maoh> derived from pAOM for tdTomato expression) in which a single copy transgene was integrated at the *Rosa26* locus [11, 14]. All the animal experiments were reviewed and approved by The Institutional Animal Care and Use Committee at Tokai University (Permit Number: #121007, #132013, #143037). All efforts were made to minimize animal suffering. For further details about the animal experiments see the Supporting Information.

To generate eGFP knockdown mice (double Tg mice, designated AWV^Δex^/eGFP, S3 Table), AWV^Δex^ mice were crossed with eGFP Tg mice [11]. The resulting offspring were checked for double Tgs by PCR using the primer sets M411/M412 to identify an amiR-eGFP transgene and M245/M701 to identify an eGFP transgene derived from eGFP Tg mice. Wild-type *Rosa26* alleles were also checked using the primer set M273/M274.

Genotyping for homozygous knockdown mice (produced by intercrossing hemizygous AWV^Δex^ mice in S3 Table) was performed by PCR using the primer sets M124/M274 and M274/M273.

The AWK^ex/Δex^/FLPe mosaic mice tended to display signs of possible premature death. Therefore, we employed a “humane endpoint,” which is defined as the time at which the animals were euthanized that was based on when the animals began to show clinical signs of illness (for example, reduced exploration, difficulty in obtaining food, and hunched posture). The animals were monitored daily, supplied with food on the bottom of the cage, and when they showed signs of illness, they were euthanized according to the Guidelines for the Care and Use of Animals for Scientific Purposes at Tokai University.

### Removal of extra sequences via FLPe recombining

AWV^ex^ and AWK^ex^ mice were generated using second-generation donor vectors, pAWV and pAWK, which included a “CAG-FLPe-polyA” cassette to aid in the self-removal of extra sequences that would be expected to result in AWV^Δex^ and AWK^Δex^ mice (S3 Table) [14]. Because self-removal of the extra sequences (such as the FLPe expression unit and the vector backbone) in the AWK^ex^ founder mouse did not occur, this founder was then crossed with FLPe Tg mice. The offspring derived from AWV^ex^ and AWK^ex^ mice were analyzed for the removal of extra sequences by PCR using the primer sets M070/M124 and M212/M273. The presence of the FLPe transgene from FLPe Tg mice was also checked using the primer set M632/M244.

### *In vitro* fertilization

Sperm cryopreservation was performed for an AWK^ex/Δex^/FLPe double Tg male mouse. Unfertilized oocytes isolated from super-ovulated female mice (BDF1) were subjected to *in vitro* fertilization (IVF) using these spermatozoa based on a protocol by Nakagata (2011) [21].

### FACS analysis

Cells that transiently or stably expressed amiRNA were washed twice with 1× phosphate buffered saline (PBS) and incubated with 0.05% trypsin-EDTA at 37°C in 5% CO_2_ to remove cells from a cell culture surface. After 5 min, complete medium was added to the plates to stop cell dissociation. After centrifuging at 800 rpm for 5 min, cells were resuspended in complete medium and then passed through a 35-μm mesh filter (Falcon 352235, BD Biosciences, Franklin Lakes, NJ, USA) for FACS analysis. Spleen cells prepared from eGFP knockdown mice were cleared of red blood cells by osmotic lysis, resuspended in complete medium, and then filtered as described for cultured cells.

Prepared cells were subjected to flow cytometry using a FACSCalibur (BD Biosciences, San Diego, CA) or an LSRFortessa (BD Biosciences) to assess knockdown efficiency and/or a FACSAria (BD Biosciences, San Jose, CA) to sort cells of interest. Data were analyzed using FlowJo software (Tree Star, Inc., Ashland, OR, USA).

For the set of Tg lines, the expression levels of eGFP and tdTomato were measured as the mean fluorescence intensity (MFI) by FACS analysis. The MFIs for eGFP and tdTomato were normalized by dividing the MFIs by eGFP Tg and tdTomato Tg lines, respectively. The experiments were repeated three times. We examined the difference in the normalized MFIs for eGFP and tdTomato across three AWV^Δex^ lines by analysis of variance (ANOVA) in which the effects of line and experiment were included as the independent factor variables. The two-way ANOVA was performed using R.

To evaluate the effect of removing a marker gene on knockdown efficiency, we measured the MFI of eGFP for a set of cell lines with (+MG) or without (−MG) the marker gene along with the wild-type and eGFP positive lines. The experiments were repeated six times for tdTomato marker gene, or seven times for *neo* ^*r*^ marker gene. The MFIs for eGFP were normalized by dividing the MFIs by the eGFP positive line within each experiment. The difference in the normalized MFI between +MG and −MG lines was evaluated by the paired *t*-test.

### Real-time PCR

Total RNA was isolated from transfected cells using a mirVana miRNA Isolation Kit (Ambion, Austin, TX), according to the manufacturer’s protocol. Purified RNA was then quantified using a NanoDrop 2000 (Thermo Scientific, Wilmington, DE). amiRNA cDNAs were synthesized from 10 ng of total RNA using a TaqMan microRNA RT Kit and miRNA-specific RT primer sets for target amiRNAs (eGFP123 and eGFP419) and endogenous control miRNA (sno202) (Applied Biosystems). The conditions used for cDNA synthesis were 16°C for 30 min, 42°C for 30 min, 85°C for 5 min, and 15°C pause. To quantify amiRNA, quantitative real-time PCR was performed using TaqMan small RNA Assays (Applied Biosystems), according to the manufacturer's instructions. In brief, the reaction mixture (10 μl) contained 4.5 μl of amiRNA cDNA (diluted 1:10, 1:20, 1:40, 1:80, 1:160, and 1:320 with nuclease-free water), 1× TaqMan Universal PCR Master Mix II, and a small RNA-specific TaqMan MGB probe (eGFP123, eGFP419 and sno202) (Applied Biosystems). Thermal cycling was conducted using StepOne Plus (Applied Biosystems) with an initial denaturation at 95°C for 10 min, followed by 40 cycles of 95°C for 15 s and 60°C for 1 min. The miRNA expression level was determined using the 2^−ΔΔCt^ method and normalized to sno202. Average threshold cycle (C) and SD values were determined from triplicate samples for each experiment, and were representative of at least three separate experiments.

Tissue biopsy samples (liver and muscle) that had been preserved in RNAlater (Qiagen, Hilden, Germany) were homogenized and total RNA was extracted using a mirVana miRNA Isolation Kit (Ambion), according to the manufacturer’s protocol. Quantitative real-time PCR was conducted as described above.

The mRNA levels of eGFP in the liver and kidney (primer sets EGFP214f/EGFP309r [22]) were assessed by SYBR-green-based real-time reverse transcription (RT)-PCR, with ACTB (β-actin) used as an internal control (primer sets β-Actin-S/β-Actin-AS).

### Analyses of transfected cells and knockdown mice

Fluorescent signals in transfected cells were observed using a Keyence BZ-9000 fluorescence microscope with the filter sets OP-66836 for eGFP and XF175 for tdTomato. Fluorescent signals in eGFP knockdown mice were observed using a Leica M165 FC with filter sets for GFP and/or red fluorescence. Mouse eyes were inspected with an Olympus SZX12 stereomicroscope.

## Results

### Comparative analysis of different amiR-eGFP expression architectures for gene-silencing in ES cells

**Fig 1:**
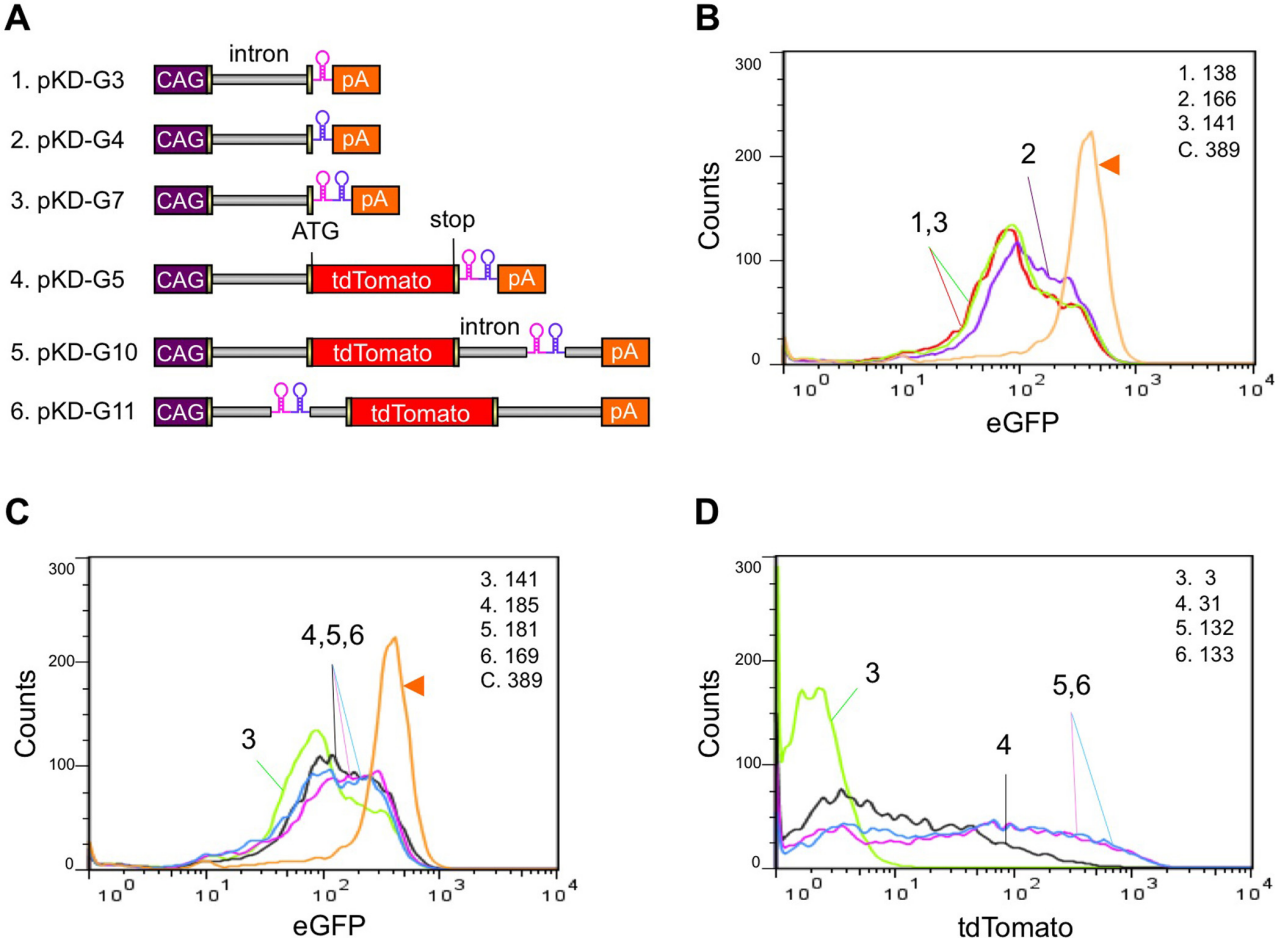
Comparison of knockdown efficiencies of different amiR-eGFP architectures in eGFP-expressing ES cells. (A) Structures of amiR-eGFP expression vectors. Two amiRNAs, each of which was designed to target different positions of the eGFP gene (hairpin structures in pink, for eGFP123, and blue, for eGFP419), were used (See S1 Table for details). (B–D) Fluorescence intensity determined after transfecting eGFP-expressing ES cells with each vector (No. 1 to 6 in A). At 2 days after transfection, the fluorescence intensity of eGFP (B and C) and tdTomato (D) was assessed by FACS. Orange lines (arrowheads in B and C) are eGFP fluorescence intensities of non-transformed eGFP-expressing ES cells (control: C). The mean fluorescence intensities (MFIs) are indicated in the upper right-hand corner of each graph in (B–D). Numbers 1 to 6 in (B–D) correspond to those shown in (A).

To establish robust knockdown system, we first investigated the optimal structure for amiRNA expression cassettes that could produce effective and desirable levels of gene knockdown. For this purpose, we generated a series of amiR-eGFP expression constructs and transfected them into eGFP-expressing ES cells to test their knockdown efficiency (Fig 1A and S2 Table). At 2 days after transfection, cells were harvested and analyzed by FACS to assess eGFP fluorescence.

Knockdown of eGFP fluorescence was detected in cells that were transfected with pKD-G3 and pKD-G4 (Fig 1B). The knockdown efficiency in pKD-G3 transfected cells was slightly greater than that of pKD-G4 transfected cells which can be due to differences in the efficiency of the amiRNA sequences (No.1 and 2 in Fig 1B). No differences in the knockdown levels were found between cells that had been transfected with either the vector that contained tandem amiR-eGFP cassettes (pKD-G7: amiR-eGFP123 and amiR-eGFP419) or the one with single amiR-eGFP cassette (pKD-G3: single amiR-eGFP123; No.1 and 3 in Fig 1B). In addition, knockdown efficiency did not seem to be influenced by either the position of amiR-eGFP in the expression construct (No. 4 to 6 in Fig 1C) or the order of two tandemly arrayed amiR-eGFP sequences (eGFP123-eGFP419 or eGFP419-eGFP123: pKD-G5 and G6; G7 and G8; G9 and G10; and G11 and G12 in S1 Fig).

In contrast, the presence of a marker gene in an expression cassette affected its knockdown efficiency. An expression cassette without the tdTomato marker gene [without marker gene (−MG)] exhibited the highest knockdown efficiency (No.3 in Fig 1C). Even though the knockdown efficiencies among all the +MG vector architectures were comparably similar (Fig 1C), the marker gene expression (tdTomato) was higher among the vectors in which the amiRNA was placed within the intron, but not in the 3′ UTR region (compare No.5 and No.6 with No.4 in Fig 1D). We observed similar effects when different experimental design (using a wild-type ES cell co-transfected with different amiRNA cassettes and an eGFP-expressing plasmid) was used (S2 Fig).

### Effects of presence of a marker gene in an amiRNA expression cassette on knockdown efficiency in stable cell lines

**Fig 2:**
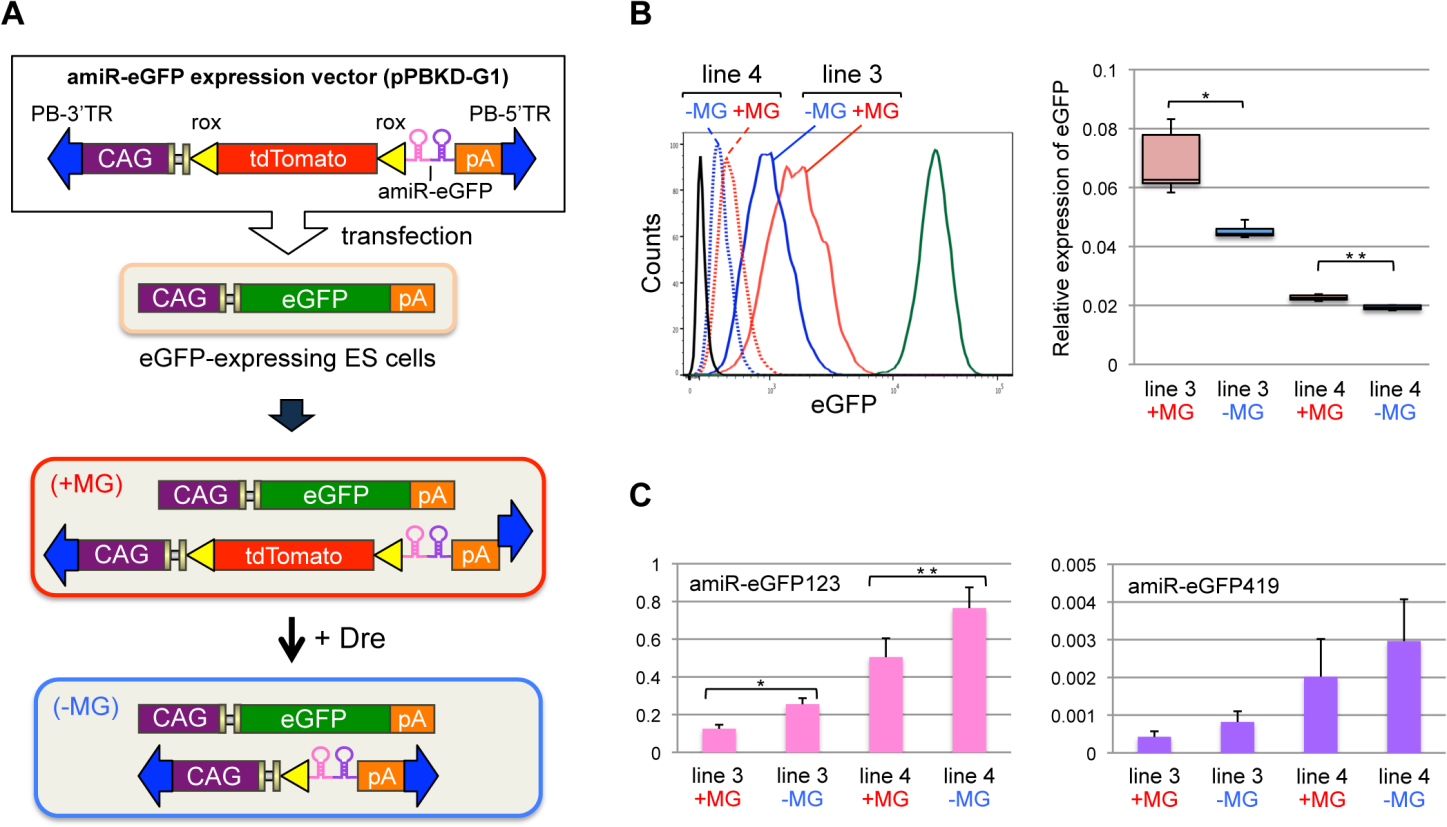
Removing the tdTomato marker gene from an amiRNA expression cassette enhances knockdown efficiency. (A) Schematic representation of the experimental strategy. An amiR-eGFP expression vector (pPBKD-G1) that contained a tdTomato marker gene flanked by *rox* sites was co-transfected into eGFP-expressing ES cells along with a pPBase and a pPBN. Stably transformed clones that exhibited tdTomato fluorescence (+MG clone) were isolated first. Then, these clones were administrated Dre recombinase to remove the tdTomato gene, which resulted in generating a marker-less clone (−MG clone). (B) eGFP fluorescence intensity in ES cells. The experiments were repeated six times and the representative histogram is shown (left). Cell lines 3 (solid line) and 4 (dot line) were used for analysis. Red and blue lines represent eGFP fluorescence in a “+MG clone” and a “−MG clone,” respectively. Green and black lines indicate eGFP fluorescence intensity in eGFP-expressing and wild-type (eGFP-negative) ES cells, respectively. The box plots show the normalized MFI obtained in six experiments (right); *P = 1.8 × 10^−3^, **P = 1.4 × 10^−5^ (paired *t*-test). (C) Quantitative real-time PCR of amiR-eGFP123 (left) and amiR-eGFP419 (right) in cell lines 3 (+MG and −MG) and 4 (+MG and −MG); The experiments were repeated three times; *P = 2.6 × 10^−3^, **P = 6.9 × 10^−3^ (two tailed Student’s *t*-test). The differences are not significant in amiR-eGFP419 expression (P > 0.05).

It is possible that the differences in knockdown efficiency in ES cells observed in previous experiments may have been simply caused by differences in transfection efficiencies among the constructs of different sizes. To assess if knockdown efficiency was indeed affected by the presence of a marker gene, we generated stable cell lines that contained an amiR-eGFP expression cassette. For this purpose, a *piggyBac* transposon system was used in combination with a Dre-*rox* site-specific recombination system (Fig 2A). This strategy allowed a well-controlled comparison scenario between a construct with a tdTomato gene and one without this gene, because both constructs were inserted at the identical genomic locus as a single-copy configuration/locus. When the *piggyBac* transposon vector pPBKD-G1 that harbored a “tdTomato and amiR-eGFP123-419” expression cassette was transfected into eGFP-expressing cells together with a pPBase, two stable cell lines were obtained (+MG; 3 and 4), each of which exhibited different tdTomato fluorescence intensity. These lines were then administrated with a Dre recombinase expression plasmid, pDre, to obtain cells without a tdTomato gene (−MG). FACS analysis of all cell lines showed that eGFP fluorescence intensity in–MG cells was lower than that in +MG cells (Fig 2B), indicating the higher knockdown of–MG architectures as observed in the previous experiment (Fig 1). The reduction was observed consistently in both the cell lines (Line 3, *P* = 1.8 × 10^−3^; Line 4, *P* = 1.4 × 10^−5^). In addition, real-time PCR analysis showed that the expression of amiR-eGFP123 in –MG cells was higher than that in +MG cells (Fig 2C), which is in agreement with the increased knockdown in –MG architectures. Even though the real-time PCR analysis for amiR-eGFP419 expression (the second part of the cassette) showed a similar trend as amiR-eGFP123, the results were not statistically significant. We obtained similar results, of higher knockdown among –MG architectures, when the tdTomato reporter marker gene was replaced with a neomycin-resistant (*neo* ^*r*^) gene (S3 Fig). Taken together, these results indicate that removing the MG, such as tdTomato or *neo* ^*r*^, from an expression cassette results in increased target gene knockdown efficiency.

### Reliable knockdown efficiency in mice generated by PITT

For reliable comparison of *in vivo* knockdown efficiency between amiRNA expression constructs, it is important to generate amiRNA Tg mice exhibiting knockdown of a target gene in a highly reproducible manner. We have established pronuclear injection-based targeted transgenesis (PITT) system that can produce Tg mice showing reproducible transgene expression [11]. We previously constructed a donor vector, pAWV (S2 Table), which harbored two amiR-eGFPs (eGFP123 and eGFP419) in the 3′ UTR of a tdTomato expression cassette in addition to the mutant *lox*Ps (*JTZ17* and *lox2272*). This vector was used to generate *Rosa26* targeted Tg mice using PITT approach as described before [14]. We obtained three amiR-eGFP (+MG) mouse lines (AWV^Δex^ lines 1 to 3, S3 Table) that were subsequently mated with eGFP Tg mice. These eGFP Tg mice used are proven to exhibit stable, reproducible, eGFP fluorescence [11]. The resulting double Tg mice (i.e., eGFP knockdown mice) were designated “AWV^Δex^/eGFP mice” (S3 Table) and were analyzed for eGFP knockdown.

**Fig 3:**
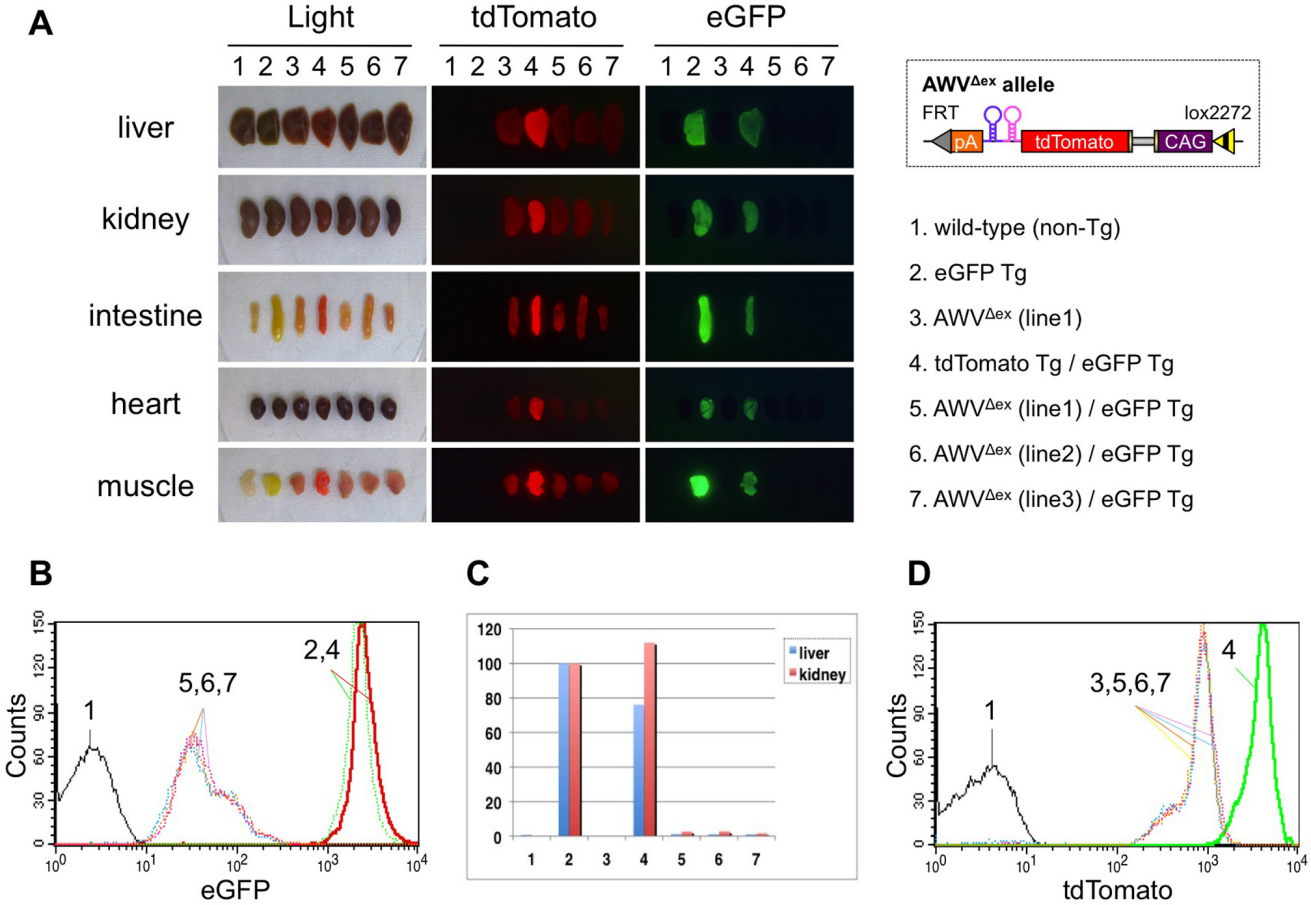
Knockdown efficiency in AWV^Δex^ mice. (A) eGFP and tdTomato fluorescent signals in mouse organs. Mouse genotypes (No. 1 to 7) are indicated on the right. Mice 5 to 7 are AWV^Δex^/eGFP mice, in which the transgene included in each mouse had been derived from a different founder (F0) mouse (lines 1 to 3). An amiRNA expression cassette in AWV^Δex^ Tg mice is shown (upper right). (B) eGFP fluorescent signals in spleen cells analyzed by FACS. (C) Quantitative real-time PCR of eGFP mRNA expression in mouse liver and kidney tissues. (D) tdTomato fluorescent signals in spleen cells analyzed by FACS. Numbers 1 to 7 in (B–D) correspond to those shown in (A).

As expected, eGFP fluorescent signals were un-detectable in any major organs, including the liver, kidney, intestine, heart, and muscle of the AWV^Δex^/eGFP mice derived from all three independent Tg lines (lines 5, 6 and 7 in Fig 3A). It is notable that eGFP fluorescence intensity in spleen cells derived from these founder lines, as analyzed by FACS, was reduced by approximately 50-fold as compared to that in spleen cells of a parental eGFP Tg mouse, and all the 3 lines exhibited similar decrease in fluorescence intensity in their spleen cells (5, 6, and 7 in Fig 3B). The efficiencies for reducing the eGFP expression were not statistically different across these three lines (*P* = 0.45). Consistent with the results obtained by FACS analysis, the eGFP mRNA levels in the liver and kidney were reduced to 1–2% compared to the parental eGFP Tg mice (5, 6, and 7 in Fig 3C). The tdTomato fluorescence intensities, that serve as a marker gene for amiRNA expression in these lines, were statistically similar to each other (*P* = 0.87). Notably, the tdTomato fluorescence intensities were lower than those in a tdTomato Tg mouse (approximately 20%). This may have been due to the placing of of amiRNAs in the 3′ UTR (but not in the intron) of an expression cassette; similar observations are reported by other groups [23, 24] (Fig 3D). Taken together, these results indicate that PITT is a useful strategy for generating reliable knockdown mice.

### Marker gene-free amiR-eGFP expressing mice are non-viable

**Fig 4:**
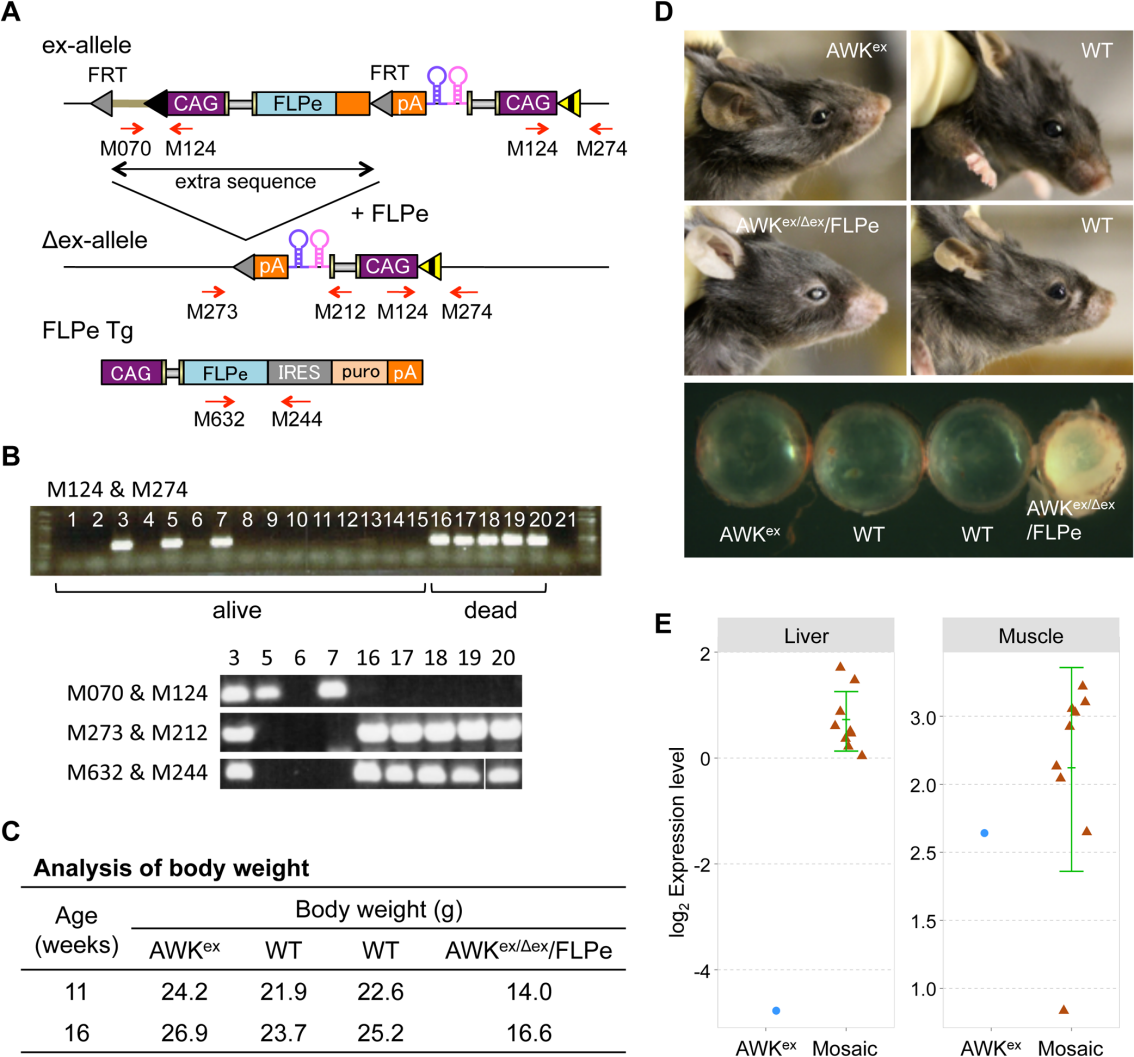
Generation of marker gene-less (−MG) amiR-eGFP-expressing mice. (A) Schematic representation of the strategy used to eliminate extra sequences in AWK mice. Extra sequences including vector sequence (shown in moss green line) and an FLPe expression cassette in the ex-allele, were removed by administering FLPe, which resulted in generating an Δex-allele. An FLPe expression cassette in FLPe Tg mice is shown below. Red arrows indicate primer-binding sites used for genotyping analysis. (B) Genotyping results of pups obtained by *in vitro* fertilization using frozen sperm derived from an AWK^ex/Δex^/FLPe mouse (lanes 1 to 20). Lane 21: negative control. PCR conducted using the primer set shown in (A) was used to assess the presence of transgenes. Fifteen individuals (1 to 15) were alive, whereas 5 pups (16 to 20) died just after birth [Note: for the sample No. 20 with primer set (M632/M244), the PCR product was loaded onto a separate lane in the same gel and rearranged in this Figure for clarity]. (C) Body weights of littermates obtained by crossing an AWK^ex^ mouse with an FLPe Tg mouse. The AWK^ex/Δex^/FLPe mouse contained both type of cells (with the AWK^Δex^ allele or AWK^ex^ allele) classified as a mosaic mouse. (D) Cataracts developed in the AWK^ex/Δex^/FLPe mouse. All mice, similar to those observed in (C), were examined at 66 weeks of age. Cataract phenotypes in additional AWK^ex/Δex^/FLPe mice are shown in S5 Fig. (E) Scatter plot of quantitative real-time PCR of amiR-eGFP123 expressions (log2 of normalized data) in nine mosaic mice and one AWK^ex^ mouse including littermates as in (D). Each circle and triangle represents an individual mouse. The values are expressed as mean ± SD represented by the green line for mosaic mice.

In our original PITT system, the F0 animals will have extra sequences that get integrated at the *Rosa26* locus (ex-allele). These sequences can be subsequently deleted by breeding the founders with FLPe-expressing mice (Δex-allele). In the second generation PITT vectors we overcome the necessity of extra breeding step by including a FLPe expression cassete that would automatically excise the extra sequence in F0 animals. We previously constructed pAWK second generation donor vector that carried a “CAG promoter - amiR-eGFP123 - amiR-eGFP419 - polyA” cassette. This vector was used to generate *Rosa26* targeted Tg mice using PITT approach (Fig 4A) [14]. Strangely, we noticed as described before that, in the pAWK vector-derived ‘amiRNA Tg’ model, the extra sequence was not deleted, even though it was derived from second generation PITT system [14]. All the F1 offspring from this founder mouse also had this extra sequence.

Next, we tried to forcefully remove this extra sequence by crossing F0 or F1 mice with FLPe Tg mice because, we thought that the presence of extra sequences, including the vector sequence and the FLPe expression cassette, could affect the expected amiRNA expression. However, we could not obtain offspring that demonstrated the successful removal of this extra sequence. Only one double Tg male (AWK^ex/Δex^/FLPe), was found to have the amiRNA expression cassette together with the FLPe transgene. Interestingly, this mouse was mosaic and contained mixture of both cells that carried an Δex-allele or an ex-allele (Fig 4A). Notably, although this mosaic mouse was fertile, it failed to transmit the amiRNA expression cassettes (either the Δex-allele or the ex-allele) to the next generation by natural mating.

Further, to obtain AWK^ex/Δex^/FLPe offspring, we performed *in vitro* fertilization (IVF) using frozen sperm prepared from the AWK^ex/Δex^/FLPe mouse. We successfully obtained 20 newborns, and 5 pups (no. 16–20 in Fig 4B) died soon after birth. Genotyping analysis of the stillborn pups showed that all were double Tg mice (AWK^Δex^/FLPe) that carried both Δex-allele and the FLPe transgene but not ex-allele (Fig 4B and S3 Table). In contrast, 3 out of the 15 living newborns (no. 3, 5, and 7 in Fig 4B) were amiR-eGFP Tg mice that contained cells with an ex-allele only or cells with both an ex-allele and an Δex-allele in a mosaic pattern (Fig 4B). Additional attempts to generate mice with the Δex-allele alone failed, while mosaic mice with cell populations with the ex-allele or the Δex-allele were obtained occasionally among the offspring of AWK^ex^ and FLPe Tg mice matings.

The mosaic mice often showed growth retardation (Fig 4C) and premature death (S4 Fig). All surviving mice (n = 9) had cataracts, which became severe as they aged (Fig 4D and S5 Fig). They also failed to transmit the transgene to the next generation (S4 Fig). Quantitative real-time PCR analysis showed that the amiRNA expression levels were higher in mosaic mice compared to the mouse with the ex-allele, as recorded in the liver samples (Fig 4E). This was in agreement with the observation that no eGFP knockdown was detected in the liver of the AWK^ex^/eGFP mice (S6 Fig). Based on all these results, we conclude that the difficulty in obtaining AWK^Δex^ mice was due to the toxicity of overexpressed amiRNA itself from the amiR-eGFP (−MG) transgene of Δex-allele.

### Failure to obtain homozygous amiRNA (+MG)-expressing mice

To further confirm whether excessive amiRNA expression adversely affects mouse survival, we attempted to generate homozygous amiR-expressing mice (+MG) by inter-crossing the hemizygous AWV^Δex^ mice described above. However, we failed to obtain homozygous AWV^Δex^ mice so far (number of wild-type: hemizygote: homozygote = 5: 7: 0). We examined the deviation of the genotype frequencies from the expected Mendelian proportions (1: 2: 1) by using a multinomial test via EMT package in R. The suggested levels of the deviation were observed (P = 0.071). Then, we evaluated whether the number of mice for homozygous AWV^Δex^ were smaller than expected (25%) by using a binomial test. We found significantly greater decrease in the numbers of homozygous mice than expected (P = 0.046). This result suggests that it is difficult to generate homozygous AWV^Δex^ mice, which is probably due to the deleterious effects on their survival by excessive amiRNA expression.

## Discussion

Most of the previously reported knockdown mouse models are generated by random integration-based transgenesis (e.g., conventional microinjection methods and virus-mediated gene transfer) and they tend to show highly variable knockdown efficiencies [25]. One of the goals of this study was to test if knockdown mice generated using PITT method show reliable expression of the knockdown cassettes. Indeed, the targeted AWV^Δex^/eGFP Tg mice, generated by PITT approach, exhibited highly reproducible knockdown efficiency. Because the expression of both “eGFP transgene” and “miRNA transgene” was very critical in this mouse model, our PITT method served as a perfect system to generate such a model since it can produce reliably expressing Tg lines. Furthermore, PITT method employs direct microinjection to target the transgene to a predetermined locus, it is less time-consuming and less expensive when compared to an ES cell-mediated method, which is typically used when one needs to generate a targeted knock-in of knockdown cassettes [10, 26]. Thus, PITT could be a powerful strategy for generating knockdown mice. We are currently developing a PITT-based strategy to generate inducible knockdown mice.

There are numerous examples of studies that attempted to obtain sufficiently high knockdown efficiency. Some studies focused on miRNA processing pathways [4, 27, 28], whereas others focused on the structures of shRNA/miRNA expression constructs [6, 29–31]. In this study, we constructed a series of expression constructs and compared their knockdown potencies. We concluded that 1) simple expression constructs that lack a marker gene (−MG) resulted in higher knockdown efficiency than those with a marker gene (+MG); and 2) knockdown efficiency was not improved/affected by bicistronic miRNA, its order, or the location of miRNA within the expression cassette. Previous studies, that used cell culture models, show both contradictory [6, 29, 30] and supporting observations [31] to our findings in this study. Notably, there are no examples of previous *in vivo* studies because, the models that allow direct comparison, like those reported here, could not be generated with other techniques with relative ease as done using PITT, and therefore our study helped draw this important conclusion.

Hu et al. reported that the presence/absence of a reporter gene had no detectable effect on RNAi efficiency [29]. They used the DsRed gene as a marker in only transiently transformed cells. In contrast, we used either tdTomato or a *neo* ^*r*^ gene as marker genes, and we examined knockdown efficiencies using both transiently transformed cells and stably transfected cells. Similar results were obtained with either of the marker genes in our experiments. In addition, we obtained consistent results both *in vitro* and *in vivo*. Considering the aforementioned factors, we are confident that the presence of a marker gene interferes with miRNA expression, at least in our experimental conditions.

Our results also suggested that the number and position of amiRNA cassettes in the transcription units had no obvious effect on knockdown efficiency, which was inconsistent in some previous studies [6, 30]. Regarding the number of amiRNAs used, our results were in agreement with those by Shan et al. [31] who showed that the knockdown efficiency obtained with three tandem amiRNAs expression cassette was at a level similar to that obtained by using the most effective amiRNA expressed by a single amiRNA expression cassette. One possibility for the contradictory results in Sun et al. [6], who used a miR-30 precursor backbone while we and Shan et al., [31] used a miR-155 precursor backbone. Future investigations will be necessary to better understand additional rules for optimal amiRNA construct design.

Grimm et al. reported that strong shRNA expression resulted in *in vivo* toxicity [7, 32] which suggest that other options, such as use of amiRNA, can be considered to avoid the pitfalls of using shRNA cassettes. However, even when amiRNA was used to produce knockdown mice, we observed, for the first time to our knowledge, *in vivo* toxicity. First, we failed to generate Tg mice with an amiRNA (−MG) transgene even though we could generate amiRNA (+MG) Tg mice. Second, homozygous knockdown mice with a marker gene (+MG) were not obtained. Because an amiRNA (−MG) expression cassette tended to transcribe large amounts of amiRNA, compared to an amiRNA (+MG) expression cassette, we concluded that our inability to produce amiRNA (−MG) Tg mice could be due to reasons such as (i) saturation effect for an endogenous microRNA pathway [7, 27, 32, 33] or (ii) excessive off-target effects of amiRNA-eGFP used in this study. If the former reason is true, then *in vivo* toxicity could be circumvented and homozygous knockdown mice could be obtained by supplying rate-limiting factors of the endogenous microRNA pathway (e.g., Exportin-5 or Argonate-2) [27, 28, 34].

The lethality of AWK^Δex^/FLPe mice was partially rescued in AWK^ex/Δex^/FLPe mosaic mice (Fig 4 and S4 Fig). In our previous reports, we showed that gene expression in PITT method generated Tg mice with an ex-allele was weak and often sporadic, whereas those with an Δex-allele showed strong and stable expression [11]. Therefore, cells with the ex-allele were considered to be amiRNA low-expression cells even when they had an amiRNA (−MG) transgene, and this transgene’s expression increased after extra sequence was removed for generating Δex-allele. The mosaic mice with both amiRNA (−MG) high-expression cells (Δex-allele) and low-expression cells (ex-allele) exhibited growth retardation and were susceptible to cataract formation. These results further strengthened our conclusion that high amiRNA expression resulted in *in vivo* toxicity.

Finally, our results showed that the presence of a marker gene in an amiRNA expression construct affected the knockdown efficiency, which could be enhanced after removing the marker gene from the expression construct. This suggests that knockdown levels can be altered by simply including or excluding a marker gene. In addition, we found that the *in vivo* toxicity of amiRNA was due to its higher level of expression. Although this problem remains to be addressed further, it is possible to express amiRNA at near highest tolerable levels in a reproducible and predictable manner by generating hemizygous amiRNA (+MG) Tg mice by our PITT method.

In most cases, RNAi-based knockdown does not completely abolish target gene expression. The previous study showed that only a 20% reduction in tumor suppressor PTEN gene expression was sufficient to show a cancer development phenotype [35]. In addition, approximately 30% of knockout mouse lines are presumed to be embryonic or perinatal lethal [36] and a large proportion of these mutants are expected to express phenotypes in heterozygotes. Considering these observations, it is highly likely that analyzing hypomorphic mice generated by our knockdown approach would be appropriate for many experimental settings.

In conclusion, this work demonstrates that (i) knockdown efficiency was increased when a marker gene from an amiRNA expression cassette was excluded, (ii) PITT-based generation of Tg knockdown mice using amiRNA cassettes without a marker gene may be difficult because of the non-tolerable levels of amiRNA expression and its toxicity in mice, (iii) the PITT can be a suitable method to generate reliable knockdown mouse models showing highest tolerable levels of amiRNA expression without any toxicity and viability issues, when a marker gene-linked amiRNA cassette is used as a heterozygous mouse.

## Supporting Information

S1 Checklist ARRIVE Guidelines Checklist. Overview and background information about the *in vivo* experiments according to the ARRIVE Guidelines.
(PDF)

S1 Fig Comparison of knockdown efficiencies of different amiR-eGFP vectors in eGFP-expressing ES cells. FACS results of all the samples analyzed in experiment shown in Fig 1. Names of the plasmids used for transfection are indicated on the upper left corner of each histogram. See S2 Table for construct details. Cont.1: pL only transfected cells; Cont.2: eGFP-expressing cells (non-transfected); Cont.3: Wild-type E14.1 ES cells (non-transfected). The MFIs are shown in the bottom corner of each graph.
(TIF)

S2 Fig Comparison of knockdown efficiencies s of different amiR-eGFP vectors in wild-type ES cells. (A) Structures of expression vectors. All amiR-eGFP expression vectors (No. 1 to 5) contained two amiR-eGFPs (amiR-eGFP123, in pink, and amiR-eGFP419, in blue). (See S2 Table for details.) (B–D) Fluorescence intensity analysis after transfecting each vector (No. 1 to 7 in A) into wild-type ES cells along with an eGFP expression vector (pG). At 2 days after transfection, eGFP and tdTomato fluorescence intensities were assessed with a fluorescence microscope (B) and by FACS (C and D). The MFIs are shown in the upper right-hand corner of each graph.
(TIF)

S3 Fig Enhanced knockdown efficiency upon removal of the neomycin resistant gene (marker) from amiRNA expression cassettes. (A) Structures of the amiRNA expression cassette and its derivatives. pPBKD-G3 and pPBKD-G2 are amiR-eGFP expression cassettes that contain a *neo* ^*r*^ gene flanked by *rox* sites. amiR-eGFPs were located at the first intron (for pPBKD-G3) or the 3′ UTR region (for pPBKD-G2) of an expression cassette. These were transfected into eGFP-expressing ES cells, which harbored an eGFP expression cassette at the *Rosa26* locus, along with a pPBase vector. After isolating stably transformed clones that exhibited G418 resistance [+MG clone; two lines were isolated for each vector (lines 3 and 8 for pPBKD-G3, and lines 1 and 2 for pPBKD-G2)], the *neo* ^*r*^ gene was removed by administering Dre recombinase, which resulted in a marker-less (-MG) clones. Red arrows indicate the primer set (M273/M838) used for genotyping shown in (B). PCR fragment size (bp) of each construct is indicated under the primer set. (B) PCR-based genotyping of the generated cell lines. eGFP: eGFP-expressing ES cell; E14.1: Wild-type E14.1 ES cells; N.C: negative control. (C) Representative histogram for eGFP fluorescence intensity in ES cell lines. Cell lines 3 (solid line) and 8 (dotted line) for pPBKD-G3-derived clones and cell lines 2 (solid line) and 1 (dotted line) for pPBKD-G2-derived clones were used for analysis. Red and blue lines indicate eGFP fluorescence in a “+MG clone” and a “−MG clone,” respectively. Green and black lines indicate eGFP fluorescence intensity in eGFP-expressing ES cells and wild-type (eGFP-negative) ES cells, respectively. The experiments were repeated seven times. *P* values (paired *t*-test) for the differences in eGFP fluorescence between “+MG clone” and “−MG clone” are shown for each line.
(TIF)

S4 Fig Representative amiR-eGFP (−MG) expressing mice. AWK^ex^ mice developed normally with normal reproductive capability. AWK^ex/Δex^ mice that had cells with the AWK^ex^ allele or AWK^Δex^ allele (mosaic mice) displayed some degree of abnormality, depending on the mosaicism (e.g., small body size and cataracts). AWK^Δex^ mice exhibited lethality immediately after birth.
(TIF)

S5 Fig Cataracts developed in the AWK^ex/Δex^/FLPe mice (#2 to #9). AWK^ex/Δex^/FLPe #9 mouse and WT mouse are littermates. Cataract phenotype in AWK^ex/Δex^/FLPe mouse (#1) that is shown in Fig 4D.
(TIF)

S6 Fig Knockdown efficiency in AWK^ex^ mice. (A) eGFP fluorescent signals in mouse organs. Mouse genotypes (No. 1 to 6) are indicated on the right.
(TIF)

S1 Table Oligos used in the present study. (XLSX)

S2 Table Vectors used in the present study. (XLSX)

S3 Table Knockdown mice (amiRNA expression mice) used in this study. (XLSX)

## References

[1] Mittal V. Improving the efficiency of RNA interference in mammals. Nat Rev Genet. 2004;5(5):355–365. 15143318 10.1038/nrg1323

[2] Podolska K, Svoboda P. Targeting genes in living mammals by RNA interference. Brief Funct Genomics. 2011;10(4):238–247. 10.1093/bfgp/elr013 21737416

[3] McBride JL, Boudreau RL, Harper SQ, Staber PD, Monteys AM, Martins I, et al Artificial miRNAs mitigate shRNA-mediated toxicity in the brain: implications for the therapeutic development of RNAi. Proc Natl Acad Sci U S A. 2008;105(15):5868–5873. 10.1073/pnas.0801775105 18398004 PMC2311380

[4] Fellmann C, Hoffmann T, Sridhar V, Hopfgartner B, Muhar M, Roth M, et al An optimized microRNA backbone for effective single-copy RNAi. Cell Rep. 2013;5(6):1704–1713. 10.1016/j.celrep.2013.11.020 24332856

[5] Zhou H, Xia XG, Xu Z. An RNA polymerase II construct synthesizes short-hairpin RNA with a quantitative indicator and mediates highly efficient RNAi. Nucleic Acids Res. 2005;33(6):e62 15805121 10.1093/nar/gni061 PMC1074311

[6] Sun D, Melegari M, Sridhar S, Rogler CE, Zhu L. Multi-miRNA hairpin method that improves gene knockdown efficiency and provides linked multi-gene knockdown. Biotechniques. 2006;41(1):59–63. 16869514 10.2144/000112203

[7] Grimm D. The dose can make the poison: lessons learned from adverse in vivo toxicities caused by RNAi overexpression. Silence. 2011;2:8 10.1186/1758-907X-2-8 22029761 PMC3234190

[8] Gao X, Zhang P. Transgenic RNA interference in mice. Physiology (Bethesda). 2007;22:161–166. 17557936 10.1152/physiol.00002.2007

[9] Hasuwa H, Kaseda K, Einarsdottir T, Okabe M. Small interfering RNA and gene silencing in transgenic mice and rats. FEBS Lett. 2002;532(1–2):227–230. 12459495 10.1016/s0014-5793(02)03680-3

[10] Premsrirut PK, Dow LE, Kim SY, Camiolo M, Malone CD, Miething C, et al A rapid and scalable system for studying gene function in mice using conditional RNA interference. Cell. 2011;145(1):145–158. 10.1016/j.cell.2011.03.012 21458673 PMC3244080

[11] Ohtsuka M, Ogiwara S, Miura H, Mizutani A, Warita T, Sato M, et al Pronuclear injection-based mouse targeted transgenesis for reproducible and highly efficient transgene expression. Nucleic Acids Res. 2010;38(22):e198 10.1093/nar/gkq860 20880997 PMC3001095

[12] Anastassiadis K, Fu J, Patsch C, Hu S, Weidlich S, Duerschke K, et al Dre recombinase, like Cre, is a highly efficient site-specific recombinase in E. coli, mammalian cells and mice. Dis Model Mech. 2009;2(9–10):508–515. 10.1242/dmm.003087 19692579

[13] Miura H, Inoko H, Inoue I, Okada Y, Tanaka M, Sato M, et al piggyBac-mediated generation of stable transfectants with surface human leukocyte antigen expression from a small number of cells. Anal Biochem. 2013;437(1):29–31. 10.1016/j.ab.2013.02.003 23416717

[14] Ohtsuka M, Miura H, Nakaoka H, Kimura M, Sato M, Inoko H. Targeted transgenesis through pronuclear injection of improved vectors into in vitro fertilized eggs. Transgenic Res. 2012;21(1):225–226. 10.1007/s11248-011-9505-y 21437715

[15] Sato M, Tanigawa M, Kikuchi N. Nonviral gene transfer to surface skin of mid-gestational murine embryos by intraamniotic injection and subsequent electroporation. Mol Reprod Dev. 2004;69(3):268–277. 15349838 10.1002/mrd.20124

[16] Hooper M, Hardy K, Handyside A, Hunter S, Monk M. HPRT-deficient (Lesch-Nyhan) mouse embryos derived from germline colonization by cultured cells. Nature. 1987;326(6110):292–295. 3821905 10.1038/326292a0

[17] Ohtsuka M, Ishii K, Kikuti YY, Warita T, Suzuki D, Sato M, et al Construction of mouse 129/Ola BAC library for targeting experiments using E14 embryonic stem cells. Genes Genet Syst. 2006;81(2):143–146. 16755138 10.1266/ggs.81.143

[18] Cadinanos J, Bradley A. Generation of an inducible and optimized piggyBac transposon system. Nucleic Acids Res. 2007;35(12):e87 17576687 10.1093/nar/gkm446 PMC1919496

[19] Wang W, Lin C, Lu D, Ning Z, Cox T, Melvin D, et al Chromosomal transposition of PiggyBac in mouse embryonic stem cells. Proc Natl Acad Sci U S A. 2008;105(27):9290–9295. 10.1073/pnas.0801017105 18579772 PMC2440425

[20] Kanki H, Suzuki H, Itohara S. High-efficiency CAG-FLPe deleter mice in C57BL/6J background. Exp Anim. 2006;55(2):137–141. 16651697 10.1538/expanim.55.137

[21] Nakagata N. Cryopreservation of mouse spermatozoa and in vitro fertilization. Methods Mol Biol. 2011;693:57–73. 10.1007/978-1-60761-974-1_4 21080274

[22] Klein D, Bugl B, Gunzburg WH, Salmons B. Accurate estimation of transduction efficiency necessitates a multiplex real-time PCR. Gene Ther. 2000;7(6):458–463. 10757018 10.1038/sj.gt.3301112

[23] Qiu L, Wang H, Xia X, Zhou H, Xu Z. A construct with fluorescent indicators for conditional expression of miRNA. BMC Biotechnol. 2008;8:77 10.1186/1472-6750-8-77 18840295 PMC2569932

[24] Du G, Yonekubo J, Zeng Y, Osisami M, Frohman MA. Design of expression vectors for RNA interference based on miRNAs and RNA splicing. FEBS J. 2006;273(23):5421–5427. 17076699 10.1111/j.1742-4658.2006.05534.x

[25] Ohtsuka M, Miura H, Sato M, Kimura M, Inoko H, Gurumurthy CB. PITT: pronuclear injection-based targeted transgenesis, a reliable transgene expression method in mice. Exp Anim. 2012;61(5):489–502. 23095812 10.1538/expanim.61.489

[26] Yu J, McMahon AP. Reproducible and inducible knockdown of gene expression in mice. Genesis. 2006;44(5):252–261. 16676321 10.1002/dvg.20213

[27] Borner K, Niopek D, Cotugno G, Kaldenbach M, Pankert T, Willemsen J, et al Robust RNAi enhancement via human Argonaute-2 overexpression from plasmids, viral vectors and cell lines. Nucleic Acids Res. 2013;41(21):e199 10.1093/nar/gkt836 24049077 PMC3834839

[28] Grimm D, Wang L, Lee JS, Schurmann N, Gu S, Borner K, et al Argonaute proteins are key determinants of RNAi efficacy, toxicity, and persistence in the adult mouse liver. J Clin Invest. 2010;120(9):3106–3119. 10.1172/JCI43565 20697157 PMC2929739

[29] Hu T, Chen P, Fu Q, Liu Y, Ishaq M, Li J, et al Comparative studies of various artificial microRNA expression vectors for RNAi in mammalian cells. Mol Biotechnol. 2010;46(1):34–40. 10.1007/s12033-010-9264-7 20300885

[30] Ely A, Naidoo T, Arbuthnot P. Efficient silencing of gene expression with modular trimeric Pol II expression cassettes comprising microRNA shuttles. Nucleic Acids Res. 2009;37(13):e91 10.1093/nar/gkp446 19474340 PMC2715259

[31] Shan ZX, Lin QX, Yang M, Deng CY, Kuang SJ, Zhou ZL, et al A quick and efficient approach for gene silencing by using triple putative microRNA-based short hairpin RNAs. Mol Cell Biochem. 2009;323(1–2):81–89. 10.1007/s11010-008-9966-3 19037714

[32] Grimm D, Streetz KL, Jopling CL, Storm TA, Pandey K, Davis CR, et al Fatality in mice due to oversaturation of cellular microRNA/short hairpin RNA pathways. Nature. 2006;441(7092):537–541. 16724069 10.1038/nature04791

[33] Khan AA, Betel D, Miller ML, Sander C, Leslie CS, Marks DS. Transfection of small RNAs globally perturbs gene regulation by endogenous microRNAs. Nat Biotechnol. 2009;27(6):549–555. 10.1038/nbt.1543 19465925 PMC2782465

[34] Yi R, Doehle BP, Qin Y, Macara IG, Cullen BR. Overexpression of exportin 5 enhances RNA interference mediated by short hairpin RNAs and microRNAs. RNA. 2005;11(2):220–226. 15613540 10.1261/rna.7233305 PMC1370710

[35] Berger AH, Knudson AG, Pandolfi PP. A continuum model for tumour suppression. Nature. 2011;476(7359):163–169. 10.1038/nature10275 21833082 PMC3206311

[36] Wong MD, Maezawa Y, Lerch JP, Henkelman RM. Automated pipeline for anatomical phenotyping of mouse embryos using micro-CT. Development. 2014;141(12):2533–2541. 10.1242/dev.107722 24850858 PMC4050698

