# Supplementary figures and images for: Assessment of Artificial MiRNA Architectures for Higher Knockdown Efficiencies without the Undesired Effects in Mice
Source: PLoS One. 2015 Aug 18;10(8):e0135919. doi: 10.1371/journal.pone.0135919 (PMC4540464; doi:10.1371/journal.pone.0135919)

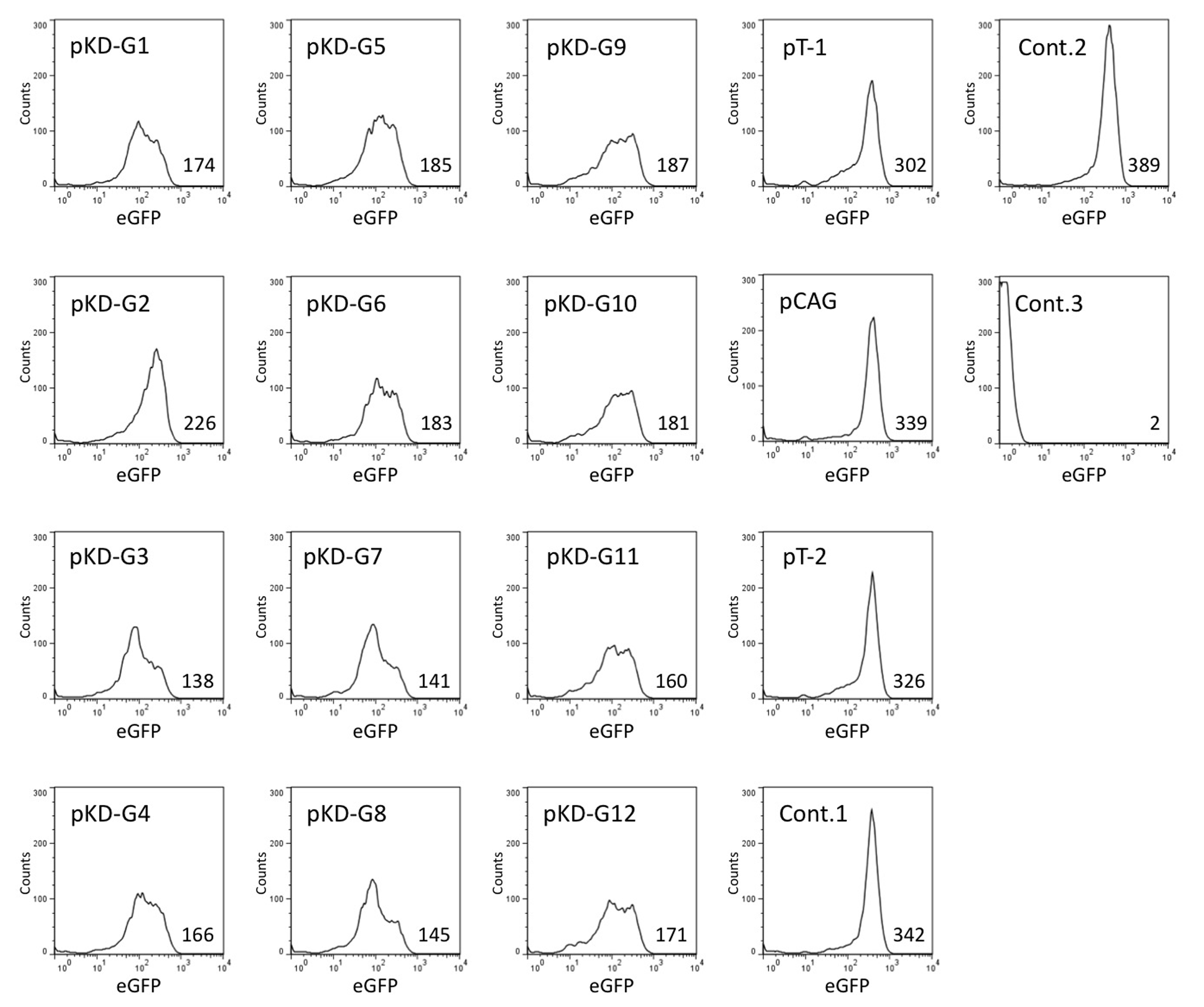

Supplement: S1 Fig — FACS results of all the samples analyzed in experiment shown in Fig 1. Names of the plasmids used for transfection are indicated on the upper left corner of each histogram. See S2 Table for construct details. Cont.1: pL only transfected cells; Cont.2: eGFP-expressing cells (non-transfected); Cont.3: Wild-type E14.1 ES cells (non-transfected). The MFIs are shown in the bottom corner of each graph. (TIF) [file pone.0135919.s002.tif]

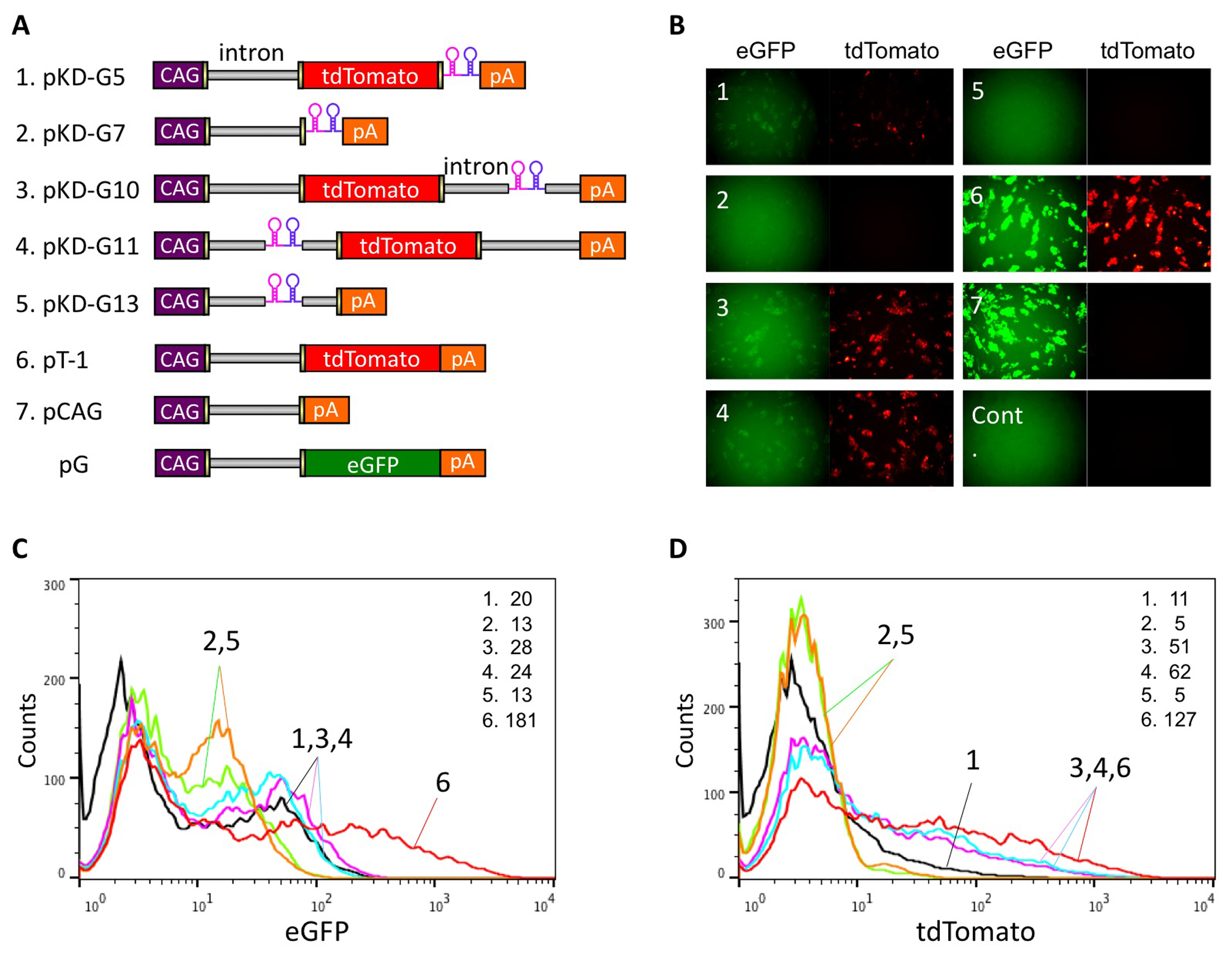

Supplement: S2 Fig — (A) Structures of expression vectors. All amiR-eGFP expression vectors (No. 1 to 5) contained two amiR-eGFPs (amiR-eGFP123, in pink, and amiR-eGFP419, in blue). (See S2 Table for details.) (B–D) Fluorescence intensity analysis after transfecting each vector (No. 1 to 7 in A) into wild-type ES cells along with an eGFP expression vector (pG). At 2 days after transfection, eGFP and tdTomato fluorescence intensities were assessed with a fluorescence microscope (B) and by FACS (C and D). The MFIs are shown in the upper right-hand corner of each graph. (TIF) [file pone.0135919.s003.tif]

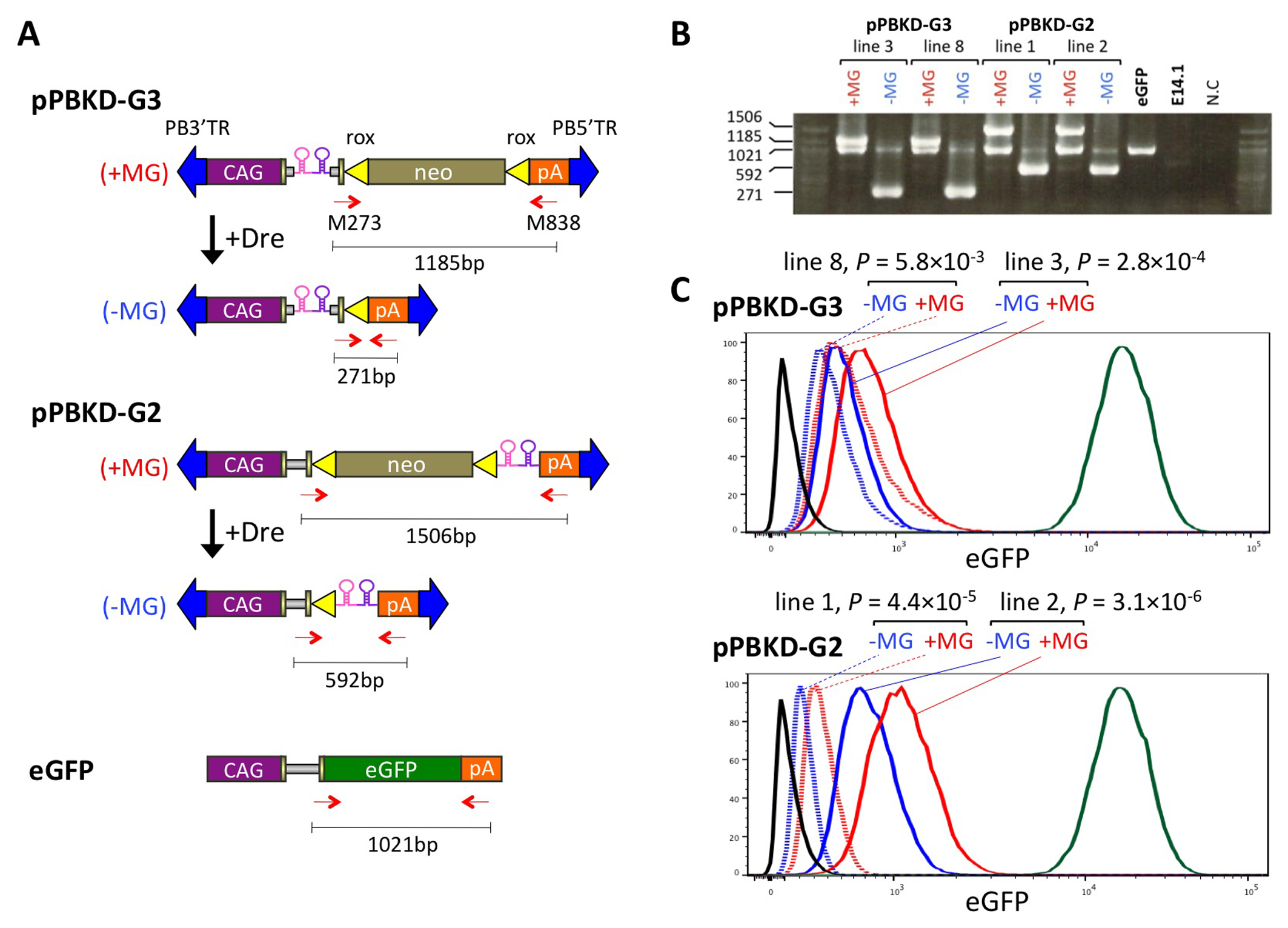

Supplement: S3 Fig — (A) Structures of the amiRNA expression cassette and its derivatives. pPBKD-G3 and pPBKD-G2 are amiR-eGFP expression cassettes that contain a neo r gene flanked by rox sites. amiR-eGFPs were located at the first intron (for pPBKD-G3) or the 3′ UTR region (for pPBKD-G2) of an expression cassette. These were transfected into eGFP-expressing ES cells, which harbored an eGFP expression cassette at the Rosa26 locus, along with a pPBase vector. After isolating stably transformed clones that exhibited G418 resistance [+MG clone; two lines were isolated for each vector (lines 3 and 8 for pPBKD-G3, and lines 1 and 2 for pPBKD-G2)], the neo r gene was removed by administering Dre recombinase, which resulted in a marker-less (-MG) clones. Red arrows indicate the primer set (M273/M838) used for genotyping shown in (B). PCR fragment size (bp) of each construct is indicated under the primer set. (B) PCR-based genotyping of the generated cell lines. eGFP: eGFP-expressing ES cell; E14.1: Wild-type E14.1 ES cells; N.C: negative control. (C) Representative histogram for eGFP fluorescence intensity in ES cell lines. Cell lines 3 (solid line) and 8 (dotted line) for pPBKD-G3-derived clones and cell lines 2 (solid line) and 1 (dotted line) for pPBKD-G2-derived clones were used for analysis. Red and blue lines indicate eGFP fluorescence in a “+MG clone” and a “−MG clone,” respectively. Green and black lines indicate eGFP fluorescence intensity in eGFP-expressing ES cells and wild-type (eGFP-negative) ES cells, respectively. The experiments were repeated seven times. P values (paired t-test) for the differences in eGFP fluorescence between “+MG clone” and “−MG clone” are shown for each line. (TIF) [file pone.0135919.s004.tif]

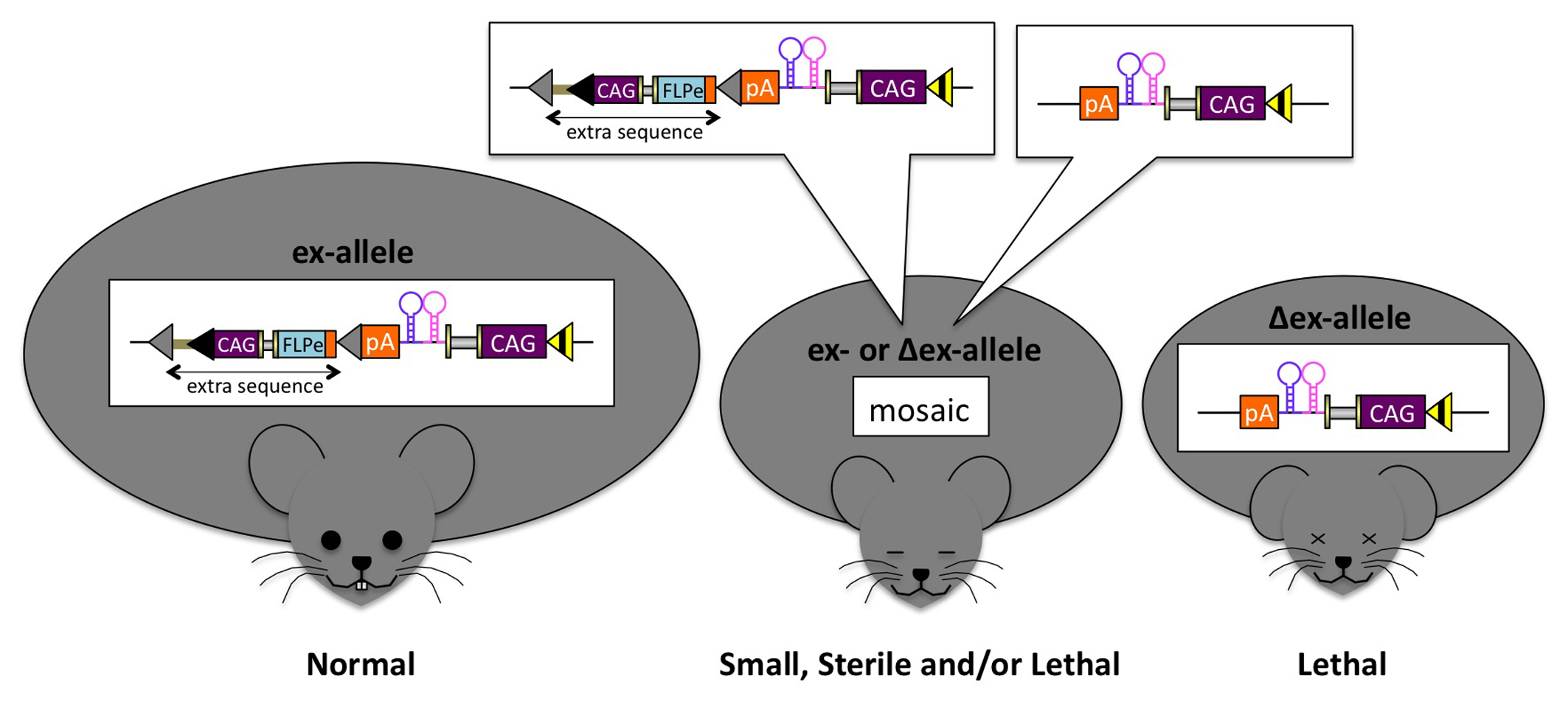

Supplement: S4 Fig — AWKex mice developed normally with normal reproductive capability. AWKex/Δex mice that had cells with the AWKex allele or AWKΔex allele (mosaic mice) displayed some degree of abnormality, depending on the mosaicism (e.g., small body size and cataracts). AWKΔex mice exhibited lethality immediately after birth. (TIF) [file pone.0135919.s005.tif]

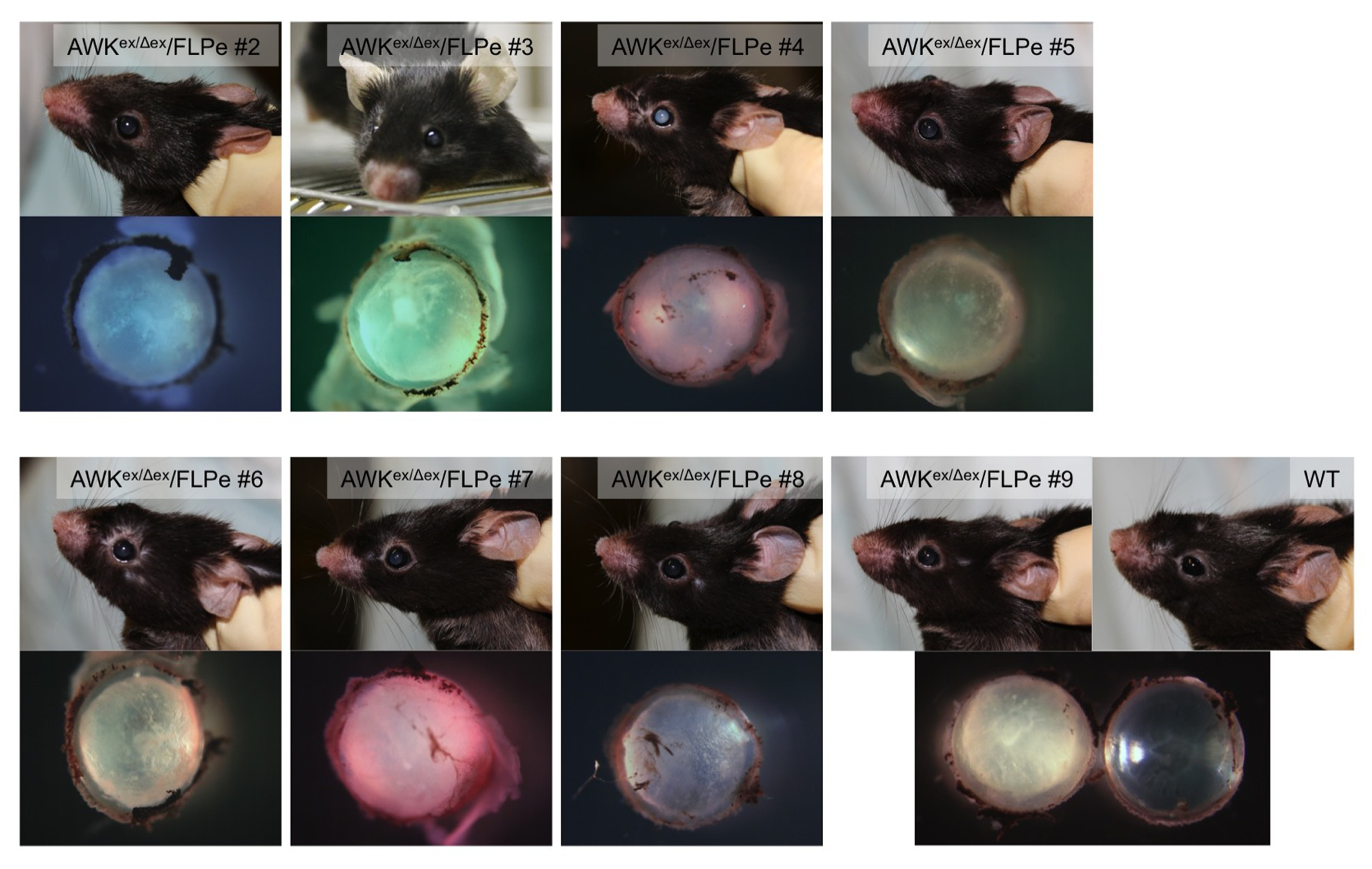

Supplement: S5 Fig — AWKex/Δex/FLPe #9 mouse and WT mouse are littermates. Cataract phenotype in AWKex/Δex/FLPe mouse (#1) that is shown in Fig 4D. (TIF) [file pone.0135919.s006.tif]

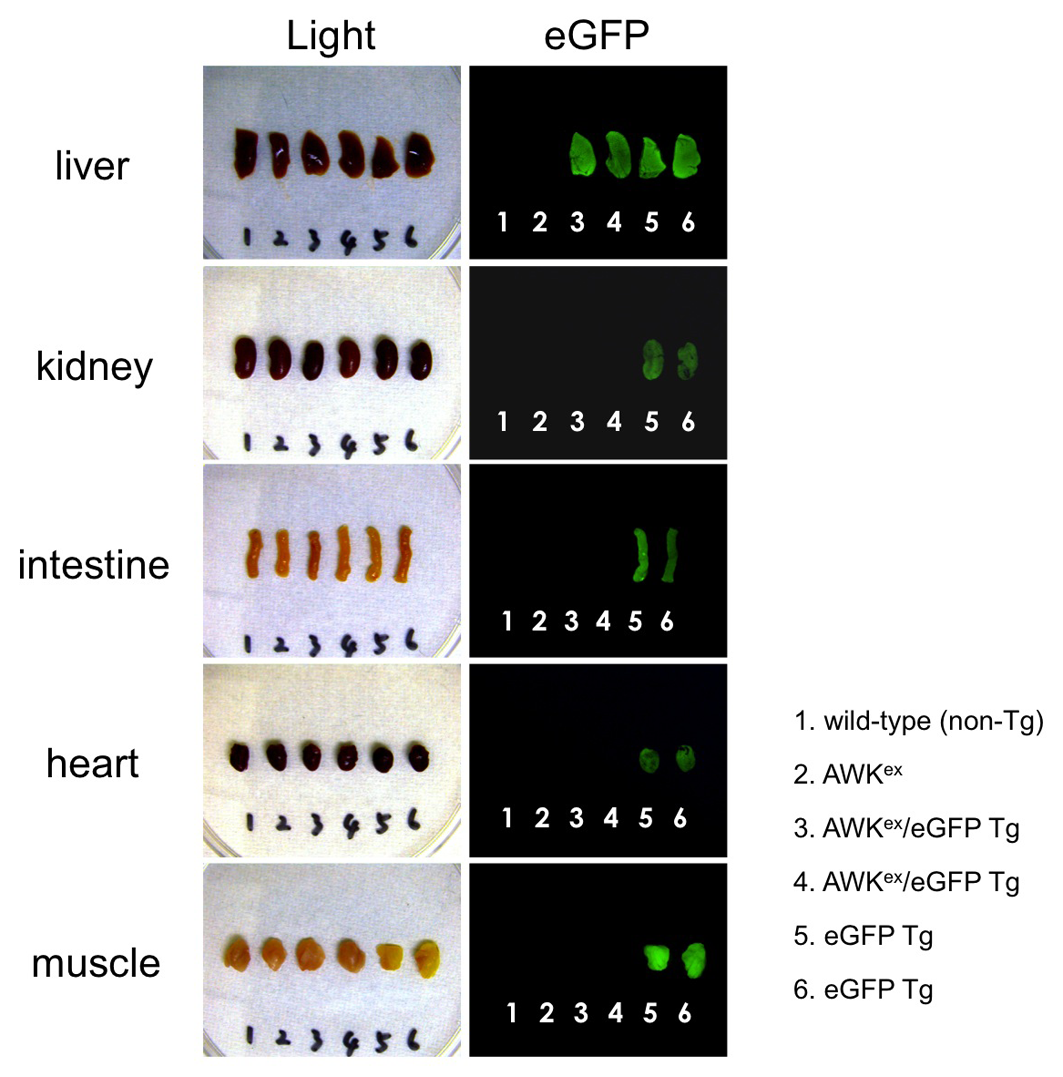

Supplement: S6 Fig — (A) eGFP fluorescent signals in mouse organs. Mouse genotypes (No. 1 to 6) are indicated on the right. (TIF) [file pone.0135919.s007.tif]
